# The neuroprotective effects of Chalcones from Ashitaba on cuprizone‐induced demyelination via modulation of brain‐derived neurotrophic factor and tumor necrosis factor α

**DOI:** 10.1002/brb3.3144

**Published:** 2023-07-04

**Authors:** Soodeh Rowhanirad, Mahnaz Taherianfard

**Affiliations:** ^1^ Division of Physiology, Department of Basic Science, School of Veterinary Medicine Shiraz University Shiraz Iran

**Keywords:** brain‐derived neurotrophic factor, Chalcones from Ashitaba, demyelination, tumor necrosis factor α

## Abstract

**Introduction:**

Multiple sclerosis (MS) is the most common demyelinating disease of the central nervous system. However, the limitations of available therapeutic strategies are frustrating, both in terms of their low efficacy and multiple side effects. Previous studies showed that natural compounds such as Chalcones possess neuroprotective effects on neurodegenerative disorders. However, few studies have so far been published on the potential effects of Chalcones on treating demyelinating disease. The present study was designed to investigate the effects of Chalcones from Ashitaba (ChA) on cuprizone‐induced noxious changes in the C57BL6 mice model of MS.

**Methods:**

The mice received normal diets (Control group: CNT), or Cuprizone‐supplemented diets either without ChA (Cuprizone group: CPZ) or with low or high (300, 600 mg/kg/day) doses of ChA (ChA‐treated groups: CPZ**+**ChA300/600). Brain‐derived neurotrophic factor (BDNF) and tumor necrosis factor alpha (TNFα) levels, demyelination scores in the corpus callosum (CC), and cognitive impairment were evaluated using the enzyme‐linked immunosorbent assay, histological, and Y‐maze tests, respectively.

**Results:**

The findings showed that ChA Co‐treatment significantly reduced the extent of demyelination in the CC and the serum and brain levels of TNFα in the ChA‐treated groups compared to the CPZ group. Besides, treatment with a higher dose of ChA significantly improved the behavioral responses and BDNF levels in the serum and brain of the CPZ**+**ChA600 group when compared with the CPZ group.

**Conclusion:**

The present study provided evidence for the neuroprotective effects of ChA on cuprizone‐induced demyelination and behavioral dysfunction in C57BL/6 mice, possibly by modulating TNFα secretion and BDNF expression.

## Graphical abstract

Our findings showed neuroprotective effects of ChA on cuprizone‐ induced deleterious changes. BDNF and TNFa could have a key role in the beneficial effects of ChA. ChA could be used as a promising treatment option for neurodegenerative disorders.

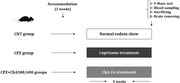

## 1 INTRODUCTION

Multiple sclerosis (MS) is the most common autoimmune inflammatory demyelinating disorder of the brain and spinal cord with a wide range of symptoms and disabilities (Compston & Coles, 2008). Despite exploring many treatment strategies for the prevention or management of MS, the patients are still suffering from progressive disabilities and the use of available therapeutic strategies is frustrating due to low efficacy and multiple side effects. However, several studies have shown the neuroprotective potential of natural compounds, such as chalcones, with therapeutic or protective effects on neurodegenerative disease (Adelusi et al., 2021).

Previous investigations showed that a Japanese herb “Ashitaba,” *Angelica keiskei*, has been used as a health‐promoting vegetable and traditional medicine (Adelusi et al., 2021; Caesar & Cech, 2016). Ashitaba contains many bioactive substances, such as chalcones. The Chalcones from Ashitaba (ChA), because of their valuable biological properties and simple chemical scaffold, are promising candidates for the treatment of neurodegenerative diseases (Rajendran et al., 2022; Zhuang et al., 2017). Small polar surface areas of these Chalcones facilitate them to cross the blood–brain barrier (BBB) and display pharmacological effects with high efficacy in protecting central nervous system (CNS) structures (Mathew et al., 2015). ChA display the protective properties and anti‐neuroinflammatory mechanisms via the downregulation of proinflammatory cytokines, such as tumor necrosis factor alpha (TNFα), inhibition of neuroinflammatory pathways, upregulation of neurotrophic factors, such as brain‐derived neurotrophic factor (BDNF), prevention of nerve senescence and BBB disruption, and synthesis of antioxidant enzymes (Adelusi et al., 2021; Tseng et al., 2019). Moreover, studies on Chalcones’ pharmacological properties, metabolism, absorption, and toxicity in mice, rats, and humans encourage their therapeutic applications (Maronpot, 2015; Nakamura et al., 2012).

Few studies have so far been published on the immunomodulatory and neuroprotective properties of synthetic or natural Chalcones derivatives. However, their underlying mechanisms of neuroprotection and effects on inflammatory responses in demyelinating disorders have been poorly explored (Chen et al., 2020; Liu et al., 2022; Mohtashami et al., 2019). Moreover, whether natural Chalcones from the Ashitaba plant may treat noxious changes induced by demyelinating agents, such as Cuprizone, as a well‐established toxic model to investigate various aspects of MS, remains unknown. Hence, the present study investigated the effects of ChA Co‐treatment on the levels of BDNF and TNFα as two key factors involved in MS, the extent of demyelination in the CC, and impairment of cognitive function in adult male C57BL/6 mice following 5 weeks of Cuprizone exposure.

## 2 MATERIALS AND METHODS

### 2.1 Animal

Previous findings showed that there were no significant gender differences in cuprizone‐mediated demyelination of the CC in C57BL/6 mice. However, this model has been extensively investigated and characterized in male C57BL/6 mice (Taylor et al., 2009; Taylor et al., 2010; Wergeland et al., 2012). Accordingly, in the current study, 20 adult male C57BL/6 mice (6‐week‐old) weighing 18–21 g (Xu et al., 2010) were obtained from the Pasteur Institute (Karaj, Iran). Procedures adopted in this study were under Shiraz University's ethical guidelines and compatible with the European Convention for protecting vertebrate animals used for experimental and other scientific purposes (95INT1M1755). The mice were housed in standard laboratory conditions (light/dark cycles of 12/12 h, room temperature of 20–24°C, and controlled humidity). All the animals had access to water and food ad libitum (Pfeifenbring et al., 2015).

### 2.2 Experimental design

**FIGURE 1:**
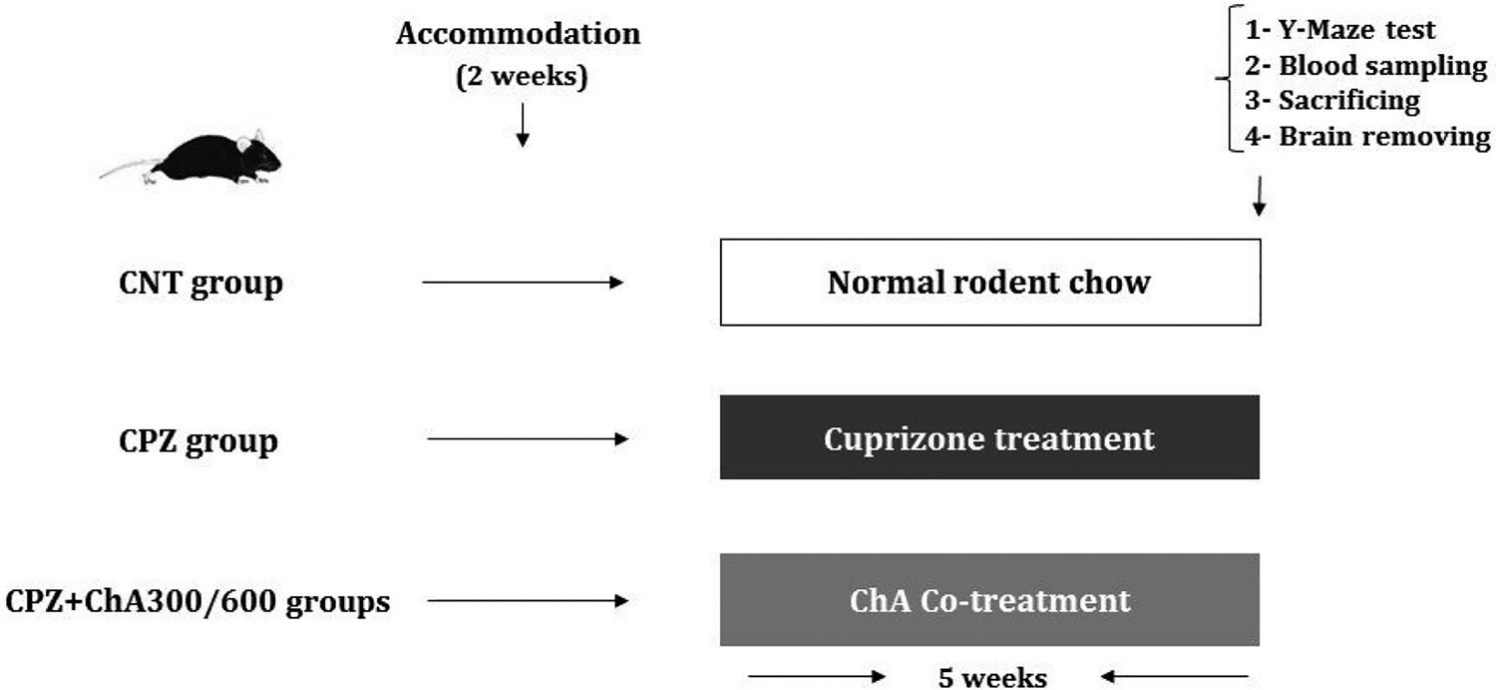
Experimental design: Four groups (*n* = 5) of animals were randomly allocated after acclimatization for 2 weeks. One group receiving a normal diet throughout the study served as control group (CNT) for each group. The CPZ group received 0.2% cuprizone mixed into the powdered normal diet for 5 weeks to induce demyelination in the CC. The two ChA‐treated (CPZ+ChA300/600) groups received 300 or 600 mg/kg/day of ChA powder which was blended into the mixture of powdered routine diet and 0.2% cuprizone for 5 weeks. All groups underwent biochemical, histological, and behavioral assessments after 5 weeks.

In the present study, the animals were randomly allocated into four groups (*n* = 5) after acclimatization for 14 days. In the control (CNT) group, intact mice were fed a routine diet. The Cuprizone (CPZ) group received 0.2% (w/w) cuprizone (finely powdered oxalic bis [cyclohexylidenehydrazide]; C9012, Sigma‐Aldrich) mixed into the powdered normal diet for 5 weeks to induce demyelination in the CC (Gingele et al., 2020; Hibbits et al., 2009). The two ChA‐treated (CPZ+ChA300/600) groups received 300 or 600 mg/kg/day of ChA powder (IACA6818, Bio Science Laboratory Co., Ltd.) (Maronpot, 2015), which was blended into the mixture of powdered routine diet and 0.2% cuprizone for 5 weeks (Figure 1). The powdered feed was freshly mixed and made every 2 days, and replaced daily. The effects of Cuprizone exposure and ChA Co‐treatment on the animal model were evaluated by biochemical, histological, and Y‐maze tests.

### 2.3 Y‐maze test

In this study, the Y‐maze test, which is widely accepted as an instrument to measure the exploring behavior and spatial working memory of rodents, was performed as described previously (Makinodan et al., 2009). Briefly, each mouse was placed at the end of one arm (34 cm long, 6 cm wide, 14.5 cm deep, and labeled A, B, or C) of the asymmetrical Y‐maze and allowed to move freely through the maze for 8 min. For each session, the total number and series of arm entries were manually recorded. The examiner was an individual blinded to the experimental groups. Actual alternation was the entries into the three arms on consecutive occasions, and the maximum alternation was the total number of arm entries minus two. Finally, the percentage of alternation was calculated as follows: Percentage of alternation = (actual alternation/ maximum alternation) ×100

### 2.4 Measurement of serum and brain levels of BDNF and TNFα

Mice were anesthetized using an I.P. injection of ketamine/xylazine (100/10 mg/kg, Sigma‐Aldrich) (Schuetze et al., 2019). Next, whole blood samples were taken from mice's hearts, the animals were decapitated, and their brains were quickly removed for dissection. For serum preparation, according to the protocol, the samples were collected in anticoagulant‐free tubes and left at room temperature for 20 min. Then, they were centrifuged (2000–3000 RPM) for 20 min, and their supernatants were collected, aliquoted, and stored at −80°C until required. Furthermore, immediately after sacrificing and dissecting animals, one of the brain hemispheres of each animal was placed on dry ice and quickly frozen at −80°C for further processing. For tissue sampling, each brain hemisphere was thoroughly homogenized (1:5, w/v) with Phosphate‐Buffered Saline (PBS) (pH: 7.4) on ice using sonication. The homogenates were centrifuged (at 2000–3000 RPM, 4°C) for 20 min, and the supernatants were carefully collected, aliquoted, and stored at −80°C until required. Finally, the serum and brain levels of BDNF and TNFα were directly assayed using specific enzyme‐linked immunosorbent assay kits under the manufacturer's instructions (Bioassay Technology Laboratory, E0013Mo, and E0117Mo, Shanghai Crystal Day Biotech Co., Ltd.).

### 2.5 Histopathology

After sacrificing the mice, the whole brain was removed. Next, the cerebrum was quickly dissected into equal right and left hemispheres on dry ice. One of these hemispheres was fixed in a 10% neutral buffered formaldehyde solution (Sigma‐Aldrich) for 72 h. The tissues were embedded in paraffin after routine tissue processing. For tissue sampling, coronal serial sections (5 μm) were prepared by a microtome. According to the literature, the CC of adult C57BL/6 wild‐type mice is the most frequently studied white matter tract in the cuprizone demyelinating model (Kipp et al., 2009; Stidworthy et al., 2003), and almost complete demyelination can be observed in this area after 5–6 weeks of cuprizone feeding (Kipp et al., 2009; Skripuletz et al., 2011). Therefore, to assess the degree of demyelination in the CC, the tissue sections were stained for myelin with Luxol Fast Blue (Sigma‐Aldrich). They were then examined using a 10**×** objective lens of an Olympus BX51 microscope. Pictures were taken by an Olympus DP 20 digital camera attached to the microscope (Olympus).

### 2.6 Quantification of images

From each animal, five coronal sections were randomly chosen and five pictures per section were taken to assess myelin levels through optical density (OD). The cross‐sectional area of the CC in the brain image was quantified by calculating the myelin density of this region using the free Java image processing software ImageJ, as previously described (Bernardes et al., 2013; Madsen et al., 2016; Wang et al., 2013). Then, the levels of myelin and demyelination of the CC in the CNT group were considered equal to 100% and 0%, respectively. Accordingly, following the evaluation of myelin levels in the CC of other groups by determining the OD of their myelin, the percentage of myelin and subsequently demyelination of the CC in these groups was calculated compared to the CNT group.

### 2.7 Statistical analysis

Data were analyzed using SPSS software version 16 (IBM Co.). Multiple comparisons between the control and the other groups were carried out by one‐way ANOVA and Tukey's post hoc test. *p*‐Value ˂ .05 was considered statistically significant. All data were presented as mean ± standard error of the mean (M ± SEM).

## 3 RESULTS

### 3.1 Effect of ChA on BDNF and TNFα levels in serum and brain

**FIGURE 2:**
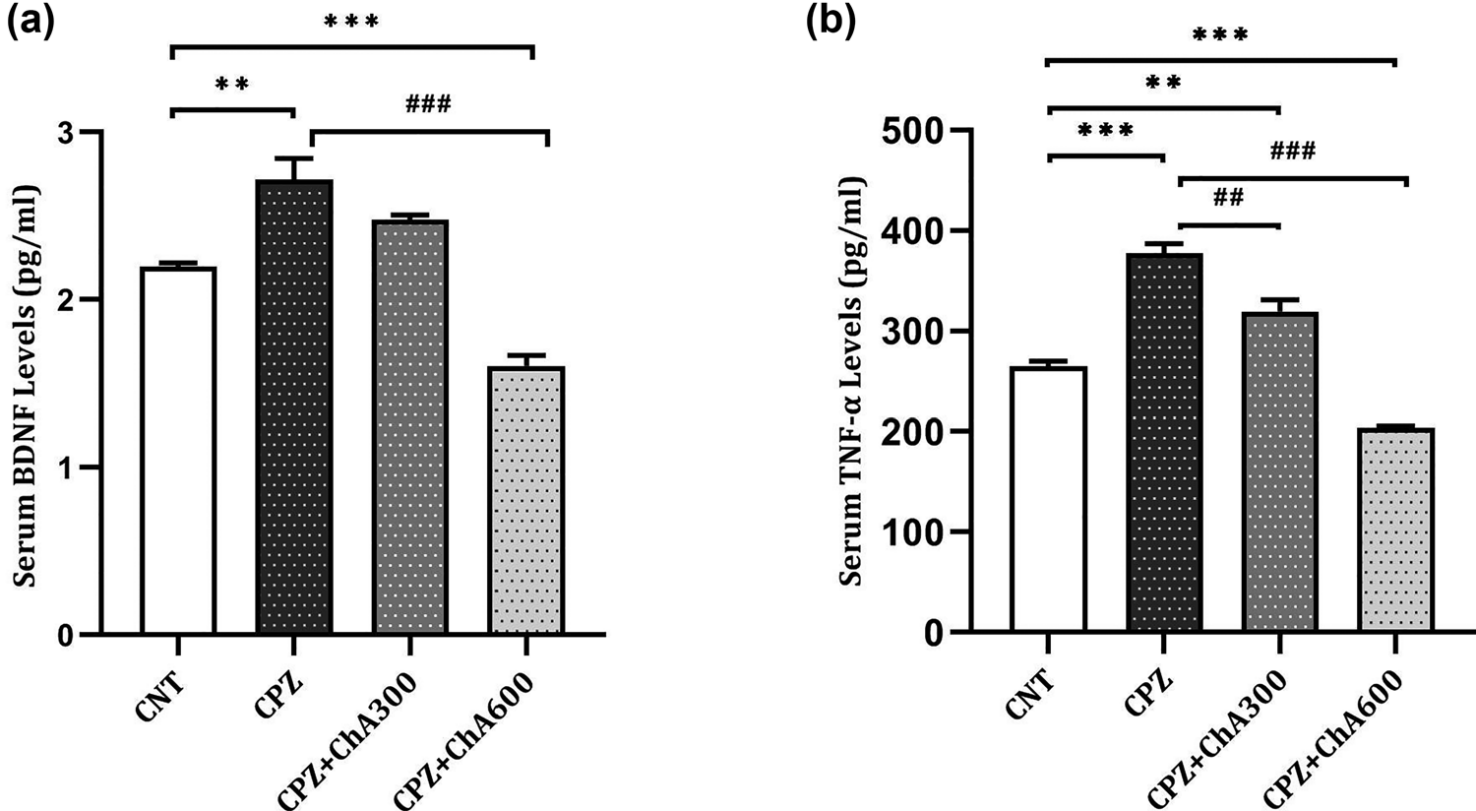
Effect of chalcones from Ashitaba (ChA) on the serum levels of BDNF and TNFα in the CNT, CPZ, CPZ+ChA300, and CPZ+ChA600 groups. ChA Co‐treatment redused Cuprizone‐induced increase in the serum levels of BDNF (a) and serum levels of TNFα (b). Tukey's multiple comparison test one‐way ANOVA (**: *p* < .01 and ***: *p* < .001 compared to the CNT group. ##: *p* < .01 and ###: *p* < .001 compared to the CPZ group). Data were shown as Mean ± SEM.

**FIGURE 3:**
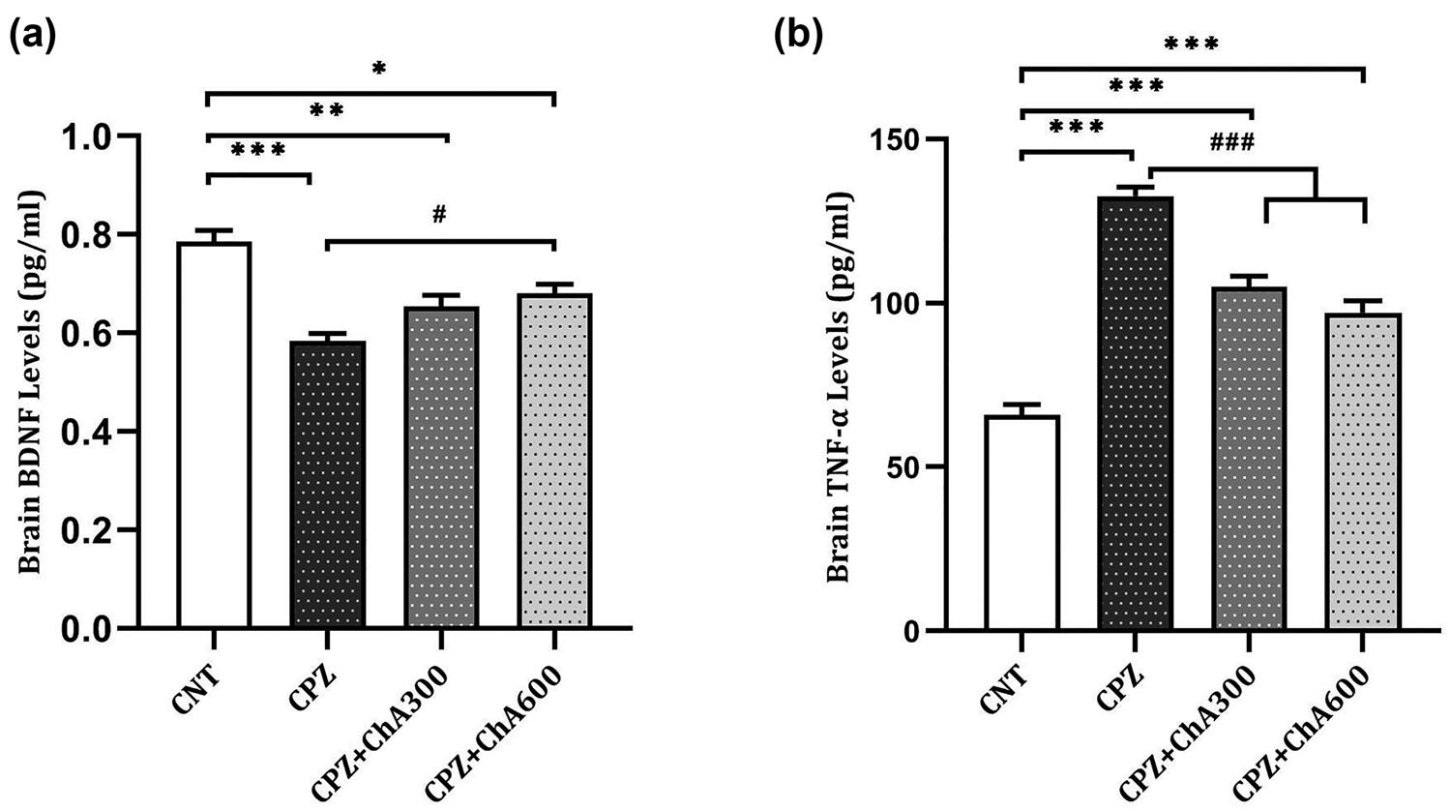
Effect of chalcones from Ashitaba (ChA) on the brain levels of BDNF and TNF‐α in the CNT, CPZ, CPZ+ChA300, and CPZ+ChA300 groups. ChA Co‐treatment reverted cuprizone‐induced decrease in the brain levels of BDNF (a). ChA Co‐treatment reverted cuprizone‐induced increase in the brain levels of TNFα (b). Tukey's multiple comparison test one‐way ANOVA (*: *p* < .05, **: *p* < .01, and ***: *p* < .001 compared to the CNT group. #: *p* < .05 and ###: *p* < .001 compared to the CPZ group). Data were shown as Mean ± SEM.

Cuprizone‐induced demyelination is associated with changes in several proinflammatory cytokines and neurotrophic factors (VonDran et al., 2011). To investigate the protective effects of ChA Co‐treatment on adverse effects induced by cuprizone exposure, BDNF and TNF‐α levels were evaluated in the serum samples and homogenous brain tissues of the animal model (shown in Figures 2 and 3).

The present study demonstrated that cuprizone exposure for 5 weeks led to significantly higher serum levels of BDNF in the CPZ (2.71 ± 0.12 pg/mL, *p* = .001) group compared to the CNT (2.19 ± 0.021pg/mL) group. Moreover, treatment with the lower dose of ChA did not cause a significant difference among the CPZ**+**ChA300 (2.47 ± 0.03 pg/mL) group compared to the CPZ (*p* = .145) and CNT (*p* = .078) groups. On the contrary, treatment with the higher dose of ChA significantly decreased the levels of serum BDNF in the CPZ**+**ChA600 (1.60 ± 0.06 pg/mL) group in comparison with both the CPZ (*p* < .000) and CNT (*p* < .000) groups (shown in Figure 2a).

On the other hand, cuprizone feeding significantly increased the serum levels of TNFα in the CPZ (377.33 ± 9.65 pg/mL, *p* < .000) and CPZ**+**ChA300 (319.49 ± 11.70 pg/mL, *p* = .002) groups compared to the CNT (264.22 ± 5.38 pg/mL) group. Interestingly, treatment with the higher dose of ChA significantly reduced the serum TNFα levels in the CPZ**+**ChA600 (203.60 ± 1.93 pg/mL) group compared to both the CPZ (*p* < .000) and CNT (*p* = .001) groups (shown in Figure 2b).

Besides, to explore how ChA Co‐treatment may impact brain levels of these factors in the cuprizone model, brain BDNF and TNFα levels were evaluated at the peak of demyelination at 5 weeks. Our results showed that cuprizone treatment caused a significant decrease in the brain levels of BDNF in the CPZ (0.58 ± 0.015 pg/mL, *p* < .000), CPZ**+**ChA300 (0.65 ± 0.02 pg/mL, *p* = .003), and CPZ**+**ChA600 (0.68 ± 0.01 pg/mL, *p* = .017) groups compared to the CNT (0.78 ± 0.02 pg/mL) group. However, the ChA Co‐treatment induced a significant increase in the brain BDNF levels of the CPZ**+**ChA600 (*p* = .024) group when compared with the CPZ group (shown in Figure 3a).

Furthermore, following cuprizone exposure, the brain levels of TNFα detected in the CPZ (132.60 ± 2.73 pg/mL, *p* < .000), CPZ**+**ChA300 (104.94 ± 3.25 pg/mL, *p* < .000), and CPZ**+**ChA600 (97.07 ± 3.60 pg/mL, *p* < .000) groups were significantly higher as compared to the CNT (65.90 ± 3.18 pg/mL) group. However, the ChA Co‐treatment caused a significant decrease in the brain levels of TNFα in both the CPZ**+**ChA300 (*p* < .000) and CPZ**+**ChA600 (*p* < .000) groups compared to the CPZ groups (shown in Figure 3b).

The findings in the current study indicated positive effects of ChA Co‐treatment on reverting the cuprizone‐induced changes in the serum and brain levels of BDNF and TNFα.

### 3.2 Effect of ChA on demyelination of the CC

**FIGURE 4:**
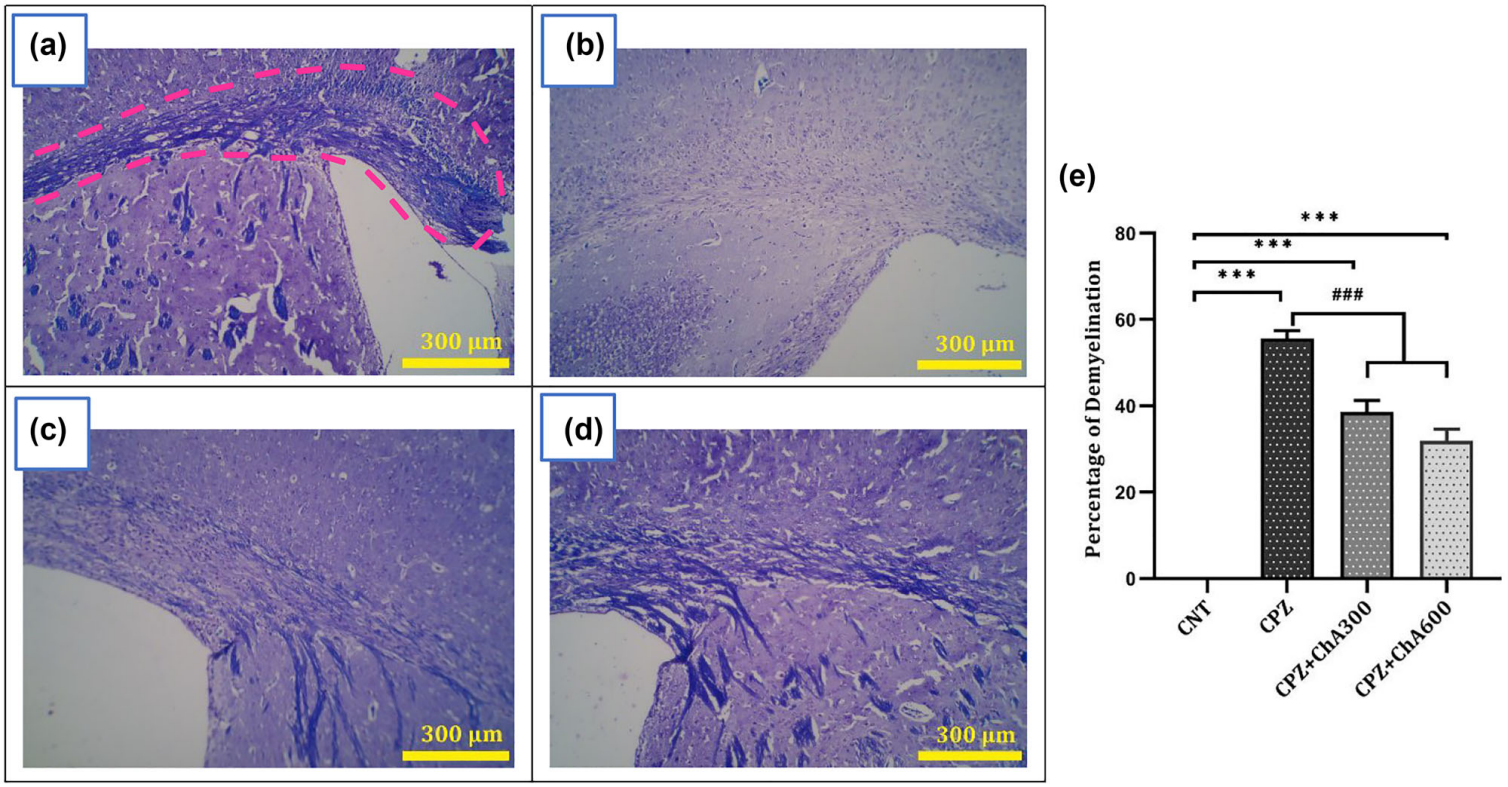
Effect of chalcones from Ashitaba (ChA) on demyelination of the corpus callosum (CC) in the control (CNT) group, intact mice; the Cuprizone‐treated (CPZ) group received 0.2% cuprizone for 5 weeks; and two ChA‐treated (CPZ+ChA300/600) groups received 0.2% cuprizone + ChA 300 or 600 mg/kg/day for 5 weeks (a–e). The photomicrographs show representative images of the CC in coronal brain sections of all groups, depicting demyelination by Luxol Fast Blue (LFB) staining, that represent the myelination status in a hemisphere of the CNT, CPZ, CPZ+ChA300, and CPZ+ChA600 groups (a, b, c, d, respectively). Blue color represents the myelination. The pink dashed line represents the border of the CC in a hemisphere (shown in “a”). Original magnification = X100. Scale bar = 300 μm for (a–d). Percentage of demyelination within the investigated area of the groups (e). Compared with the CNT group, myelination is significantly reduced after 5 weeks of cuprizone treatment. ChA co‐treatment improved myelination levels in the ChA‐treated groups. Tukey's multiple comparison test one‐way ANOVA (***: *p* < .001 compared to the CNT. ###: *p* < .001 compared to the CPZ). Data were shown as Mean ± SEM.

The extent of demyelination in the CC of all groups with different treatment paradigms was evaluated using LFB staining. Representative brain sections of the groups are presented in Figure 4. The CC of the CNT group was fully myelinated (0.00 ± 0.00%), while the intake of 0.2% cuprizone for 5 weeks resulted in significant demyelination in the CC of the CPZ (55.61 ± 1.85%, *p* < .000), CPZ**+**ChA300 (38.58 ± 2.72%, *p* < .000), and CPZ**+**ChA600 (31.97 ± 2.66%, *p* < .000) groups when compared with the CNT group. Nonetheless, the ChA Co‐treatment significantly decreased demyelination in the CC of the CPZ**+**ChA300 (*p* < .000) and CPZ**+**ChA600 (*p* < .000) groups compared to the CPZ group (shown in Figure 4). The results suggested a protective action of ChA against cuprizone‐induced demyelination, which was seen along with the improvement in the serum and brain levels of BDNF and TNFα in the ChA‐treated groups.

### 3.3 Effect of ChA on behavioral responses

**FIGURE 5:**
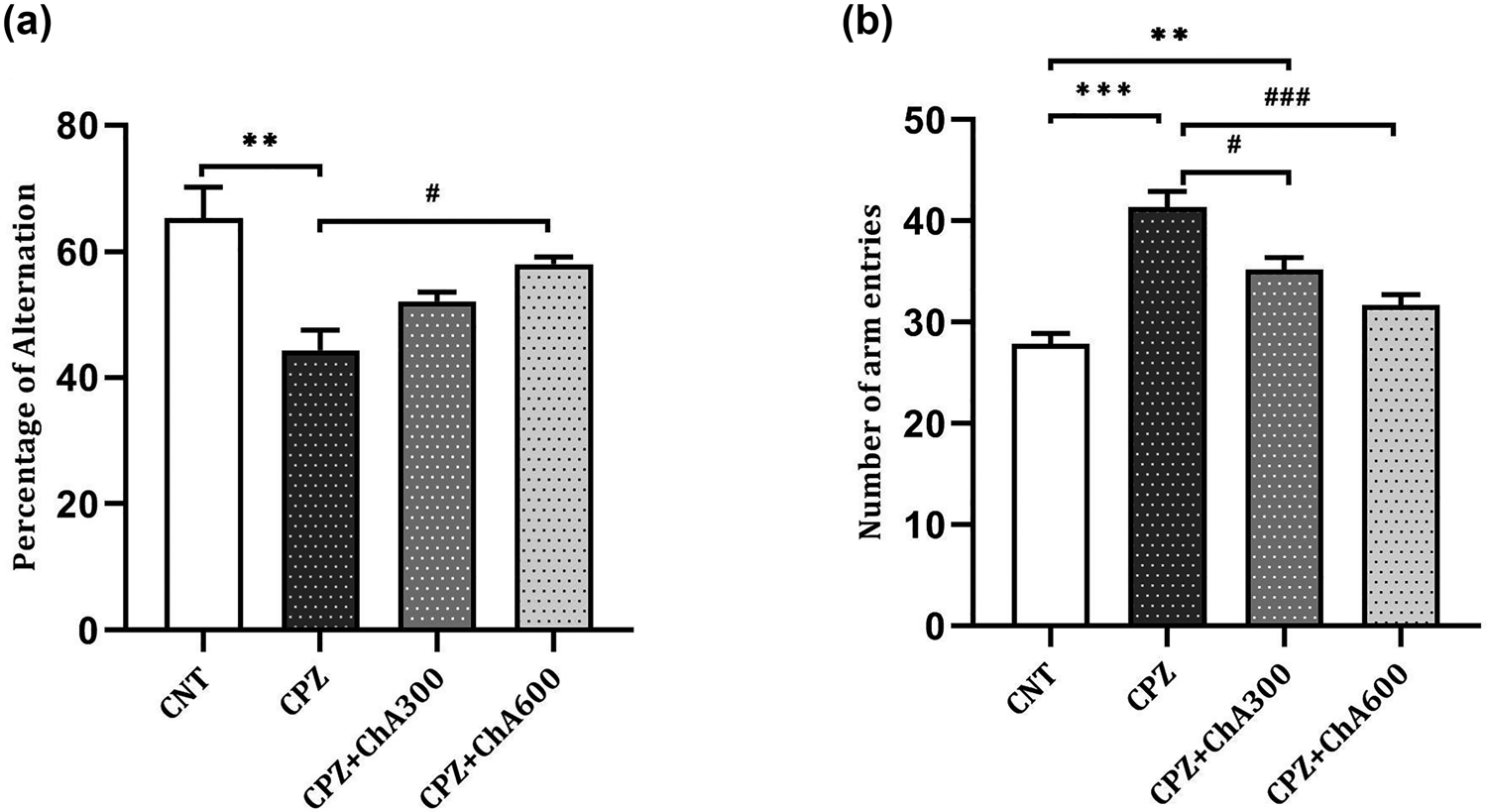
Effect of chalcones from Ashitaba (ChA) on cuprizone‐induced behavioral changes in the Y‐maze test after 5 weeks. ChA Co‐treatment ameliorated cuprizone‐induced decrease in the alternation of behavior (a). ChA Co‐treatment ameliorated cuprizone‐induced increase of arm entries (b). Tukey's multiple comparison test one‐way ANOVA (**: *p* < .01 and ***: *p* < .001 compared to the CNT group. #: *p* < .05 and ###: *p* < .001 compared to the CPZ group). Data were shown as Mean ± SEM.

To explore the behavioral outcome of cuprizone feeding for 5 weeks, with or without ChA treatment, we performed the Y‐maze test (Figure 5). Evaluation of working memory using the Y‐maze test demonstrated that cuprizone exposure without ChA treatment significantly decreased the alternation behavior of the Y‐maze test in the CPZ (44.33 ± 3.17%, *p* = .005) group in comparison to the CNT (65.33 ± 4.91%) group. However, the ChA‐treated groups, which were fed with cuprizone and ChA, were not significantly different when compared to the CNT group. Moreover, treatment with ChA significantly improved the alternation behavior in the CPZ**+**ChA600 group as compared to the CPZ (*p* = .049) group (shown in Figure 5a).

Moreover, cuprizone treatment induced a significant increase in the number of arm entries of the Y‐maze test in the CPZ group (41.33 ± 1.58, *p* < .000) compared to the CNT (27.83 ± 1.07) group. However, the ChA Co‐treatment caused a significant decrease in the number of arm entries in the CPZ**+**ChA300 (*p* = .012) and CPZ**+**ChA600 (*p* < .000) groups compared to the CPZ group. Besides, no significant difference was observed between the CPZ**+**ChA600 (31.67 ± 1.05, *p* = .171) and the CNT groups (shown in Figure 5b). In the present study, ChA Co‐treatment improved the working memory deficit caused by cuprizone feeding in parallel with reducing the degree of demyelination and reverting the levels of BDNF and TNFα in the ChA‐treated groups. These results suggested the promising potential of ChA as a therapeutic agent for demyelinating diseases.

## 4 DISCUSSION

Natural compounds such as chalcones have been subjects of increased interest due to their therapeutic potential in neurodegenerative diseases (Adelusi et al., 2021; Oh et al., 2013). However, their underlying mechanisms of neuroprotection and effects on inflammatory responses in demyelinating disorders have been poorly explored (Adelusi et al., 2021; Yasuda et al., 2014). In the present study, we demonstrated the immunomodulatory effects of Chalcones from Ashitaba on regulating BDNF and TNFα levels in the serum and brain. We also detected the protective effect of ChA on the extent of demyelination in the CC, and their ameliorative effect on cognitive impairments in the cuprizone mice model. ChA may, therefore, have neuroprotective effects on the toxic demyelinating model based on the present findings. However, further investigations are needed to discover several mechanisms of ChA actions in the protective processes.

MS is an autoimmune inflammatory demyelinating disease of the CNS with heterogeneous clinical manifestations, including demyelination and inflammation in brain lesions, involvement of proinflammatory cytokines and neurotrophic factors, and deficits in cognitive functions (Patanella et al., 2010). In the present study, we used the cuprizone model, as a well‐established demyelinating model to investigate various aspects of MS.

Evidence indicates that inflammatory cytokines interact with neurotrophins in the CNS. Moreover, upregulation of proinflammatory cytokines, such as TNFα, can alter BDNF levels and promote neurodegeneration, which in turn can cause cognitive impairment. Therefore, previous studies investigated the levels of TNFα and BDNF, as two key factors involved in demyelination (Patanella et al., 2010; Rowhani‐Rad & Taherianfard, 2019). The present study showed that cuprizone‐induced demyelination led to significantly higher levels of serum TNFα and BDNF in the CPZ group. Consistent with the current results, Schmitz and Chew's investigations (2008) indicated that cuprizone exposure led to the production and release of TNFα by activated immune cells (T‐lymphocytes, macrophages/monocytes, as well as glial cells) (Schmitz & Chew, 2008). Additionally, some T‐helper (Th) cells, which were specific for myelin autoantigens, secreted BDNF following demyelination and antigen stimulation (Peixoto et al., 2015). On the other hand, it was previously demonstrated that the serum levels of BDNF and TNFα were both altered in parallel in animals exposed to cuprizone (Rowhani‐Rad & Taherianfard, 2019). Besides, it was observed that the higher levels of TNFα induced the secretion of BDNF through immune system cells, particularly activated T lymphocytes (Clarner et al., 2015). This suggests that TNFα serves as a mediator for the secretion of BDNF in response to cuprizone exposure. However, our results showed that treatment with the higher dose of ChA not only reverted the basal levels but also further reduced the concentrations of the serum BDNF and TNFα, reflecting a mechanism to compensate for the cuprizone‐mediated upregulation of these factors. The present findings are consistent with those of previous studies, which showed that Chalcones exhibit a broad spectrum of pharmacological activities. The Chalcones possess immunomodulatory effects on T cells through induction of apoptosis in the activated T lymphocytes, and inhibition of responses, proliferation, and production of Th cells, which lead to suppressing the secretion of the proinflammatory cytokines, including TNFα. According to literature, Chalcones inhibit the PI3K/AKT/GSK‐3β/NF‐κβ signaling pathway leading to downregulation of the NF‐κB‐dependent gene products, such as TNFα, and suppressing the inflammation induced by this factor (Caesar & Cech, 2016; Fontes et al., 2014; Funakoshi‐Tago et al., 2009; Lee et al., 2015). Moreover, these compounds lead to the prevention of neuronal death and protection of the BBB (Zhang et al., 2018). In addition, Chalcones can modulate the JAK2/STAT3 signaling pathway, as a crucial pathway to inflammation by promoting the release of cytokines, such as TNF‐α. Therefore, inhibition of the JAK2/STAT3 axis represents an anti‐inflammatory and neuroprotective strategy that can be obtained by these compounds. Overall, Chalcones represent neuroprotective effects, which may occur via their ability to modulate inflammation (Adelusi et al., 2021).

Besides, evidence revealed that the chalcones from Ashitaba suppress the production of BDNF increased by activated Th cells (Kumar et al., 2013). Furthermore, the immune system cells seem to be the target of neurotrophin autocrine and paracrine actions by producing and releasing neurotrophins and expressing neurotrophin receptors (Caggiula et al., 2005). Thus, BDNF modulates and decreases TNFα levels, which leads to reduced BDNF levels through these cells (VonDran et al., 2011). Therefore, the present findings demonstrated the serum levels of BDNF and TNFα could be regulated through the modulatory mechanisms of chalcones affecting the several pathways and the interactions between these two factors.

On the other hand, in the current study, cuprizone treatment significantly decreased BDNF brain levels in cuprizone‐exposed mice. The results are consistent with the findings of previous studies, which indicated that although glial cells increase BDNF expression following an injury, demyelination itself reduces the brain levels of BDNF through various mechanisms (Fulmer et al., 2014; VonDran et al., 2011). Besides, an intact BBB has been reported in the cuprizone mouse model (Praet et al., 2014), and BDNF transport is negligible through the BBB (Pilakka‐Kanthikeel et al., 2013), suggesting that serum BDNF levels may not reflect BDNF levels in the brain. In contrast, our findings indicated that cuprizone treatment induced a significant increase in brain TNF‐α levels, which is in agreement with previous studies that showed elevated brain levels of TNF‐α along with neuroinflammation in brain lesions following cuprizone ingestion (Elbaz et al., 2018; Voss et al., 2012). Altogether, our results showed that the concentrations of BDNF and TNFα were altered inversely in the brain tissues of the cuprizone‐treated groups that are consistent with previous studies (Rowhani‐Rad & Taherianfard, 2019).

However, Co‐treatment with the higher dose of ChA significantly reverted the concentrations of these factors in the CPZ**+**ChA600 group. The present results agree with the previous findings, which showed that the Chalcones administration reduces proinflammatory mediators, including TNFα, by suppressing the activations, productions, and responses of T lymphocytes (Fontes et al., 2014; Funakoshi‐Tago et al., 2009; Lee et al., 2015). Besides, these compounds inhibit the PI3K/AKT/GSK‐3β/NF‐κβ and JAK2/STAT3 signaling pathways leading to the downregulation of cytokines, such as TNF‐α. Therefore, Chalcones may induce anti‐inflammatory and neuroprotective properties through these mechanisms (Adelusi et al., 2021; Zhang et al., 2018).

On the other hand, previous investigations indicated that BDNF, a main product of CREB‐mediated transcription (Peixoto et al., 2015), was affected by ChA. Chalcones‐mediated modulation of the CREB/BDNF/Bcl2 axis leads to the sustained expression of BDNF via the upregulation of the CREB‐BDNF pathway and the survival of neuronal cells via the Bcl2‐antiapoptotic family (Kumar et al., 2013; Lee et al., 2018; Lv et al., 2016). Therefore, our results are in agreement with former studies that showed that Chalcones ameliorate neurodegenerative diseases through critical mechanisms leading to the induction of neurotrophic factors such as BDNF and inhibition of proinflammatory cytokines such as TNFα in the brain (Adelusi et al., 2021; Fontes et al., 2014; Funakoshi‐Tago et al., 2009; Lee et al., 2015).

In order to elucidate the protective effects of Chalcones on cuprizone‐induced demyelination, we evaluated the extent of demyelination in the CC of the animals. The current results showed that cuprizone‐exposed mice had extensive demyelination in the CC, 5 weeks following the exposure. These results agree with other studies that determined the effects of cuprizone treatment. According to previous studies, cuprizone feeding causes oxidative stress, which contributes to the apoptosis of oligodendrocytes, activation of microglia and astrocytes, the release of proinflammatory cytokines, and degeneration of myelin sheaths a few days following initiation of the exposure. These events lead to inflammation and demyelination in brain lesions (Atanasov et al., 2021; Praet et al., 2014). In addition, cuprizone treatment (0.2%) for 5 weeks induces extensive demyelination in the CC of C57BL6 mice (Gingele et al., 2020; Kipp et al., 2009; Skripuletz et al., 2011). However, our results indicated that ChA Co‐treatment led to a significant decrease in the extent of demyelination in the CC of the ChA‐treated groups. Consistent with the present study, evidence showed that these compounds present therapeutic properties, including anti‐oxidative and anti‐inflammatory effects (Atanasov et al., 2021; Calderon‐Montano et al., 2011; Ohnogi et al., 2012), which have been linked to their neuroprotective effects (Sandoval et al., 2020). Besides, Chalcones increase the expression of BDNF, a neurotrophic factor essential for neuronal development and survival (Hyman et al., 1991; Kowiański et al., 2018). Moreover, an increase in the brain levels of BDNF induced higher proliferation and differentiation of oligodendrocyte progenitor cells, which could improve myelination in the CNS (Clarner et al., 2015; Fulmer et al., 2014; Tsiperson et al., 2015). Accordingly, these results demonstrated that ChA possess a protective effect on myelin sheaths and prevent the extension of demyelination in the CC of the mouse model through anti‐inflammatory and neurotrophic properties.

To find the effect of ChA on improving spatial working memory, we tested the number of spontaneous alternations and the number of arm entries in the Y‐maze test. Consistent with our results, the previous reports indicated that cuprizone‐exposed mice exhibited global and widespread demyelination in the brain. The mice displayed impairment of spatial working memory that is believed to be the direct result of demyelination (Xu et al., 2010). It was found that neuronal function deficiencies could result from myelin sheath abnormalities. Myelin is the main component for the proper functioning of neuronal circuits, and myelination is actively involved in the plasticity of neuronal circuits and is essential for cognition and motor skills (Li et al., 2015). Moreover, former researchers determined that cuprizone administration increases the brain levels of TNF‐α (Elbaz et al., 2018; Voss et al., 2012) and decreases the expression of BDNF through demyelination (Kim et al., 2019; Tsiperson et al., 2015). Indeed, BDNF plays a neuroprotective role in cognitive functions and is vital to learning, memory, and higher thinking (Patanella et al., 2010). Therefore, the lower levels of BDNF in the brain cause cognitive deficits in the animal model (Tanaka et al., 2006). However, the present findings showed that ChA Co‐treatment significantly improved the lower spontaneous alternation and higher number of arm entries induced by cuprizone in the CPZ**+**ChA600 group. The findings agree with previous studies indicating that Ashitaba compounds are effective in reverting the reduced expression of BDNF and improving short‐term memory (Oh et al., 2013). Additionally, it was revealed that Chalcones have an ameliorative effect on neurological motor disability and memory dysfunction in the Y‐maze test (Atanasov et al., 2021; Oh et al., 2013). The cognitive‐enhancing activities of these compounds might result from regulating TNFα secretion and BDNF expression (Atanasov et al., 2021). According to the current results, ChA treatment may have promising potential to improve demyelination‐induced cognitive impairment, possibly through modulating BDNF and TNFα levels, which reduces demyelination.

## 5 CONCLUSION

The current study demonstrated the neuroprotective effects of ChA on cuprizone‐induced demyelination and behavioral dysfunction in mice, possibly by modulating TNFα secretion and BDNF expression. This study suggests that ChA may have therapeutic potential in the management of demyelinating disorders and be a useful supplementary drug in neurodegenerative diseases, including MS. Further research works on ChA mechanisms of action and their neuroprotective effects on neurodegenerative disorders are awaited with great interest.

## AUTHOR CONTRIBUTIONS

Soodeh Rowhanirad and Mahnaz Taherianfard designed the experiments and Soodeh Rowhanirad performed the experiments. Soodeh Rowhanirad and Mahnaz Taherianfard analyzed the data. Soodeh Rowhanirad wrote the first draft. Mahnaz Taherianfard made the amendments and provided financial support. All authors read and approved the final manuscript. Soodeh Rowhanirad and Mahnaz Taherianfard confirm the authenticity of all the raw data. All authors have read and approved the final manuscript.

## CONFLICT OF INTEREST STATEMENT

The authors have no conflict of interest to declare.

## ETHICS STATEMENT

All experimental protocols were performed according to the Ethical Committee for Animal Experiments at Shiraz University (Animal welfare code: 95INT1M1755).

### PEER REVIEW

The peer review history for this article is available at https://publons.com/publon/10.1002/brb3.3144

## Data Availability

All data generated or analyzed during this study are included in this article. Further inquiries can be directed to the corresponding author.
